# Postinjury Complications: Retrospective Study of Causative Factors 

**DOI:** 10.2196/14819

**Published:** 2019-09-26

**Authors:** Elizabeth Warnack, Hersch Leon Pachter, Beatrix Choi, Charles DiMaggio, Spiros Frangos, Michael Klein, Marko Bukur

**Affiliations:** 1 NYU Langone Health/Bellevue Hospital Center New York, NY United States

**Keywords:** failure to rescue, error, harm, trauma

## Abstract

**Background:**

Injury care involves the complex interaction of patient, physician, and environment that impacts patient complications, level of harm, and failure to rescue (FTR). FTR represents the likelihood of a hospital to be unable to rescue patients from death after in-hospital complications.

**Objective:**

This study aimed to hypothesize that error type and number of errors contribute to increased level of harm and FTR.

**Methods:**

Patient information was abstracted from weekly trauma performance improvement (PI) records (from January 1, 2016, to July 19, 2017), where trauma surgeons determined the level of harm and identified the factors associated with complications. Level of harm was determined by definitions set forth by the Agency for Healthcare Research and Quality. Logistic regression was used to determine the impact of individual factors on FTR and level of harm, controlling for age, gender, Charlson score, injury severity score (ISS), error (in diagnosis, technique, or judgment), delay (in diagnosis or intervention), and need for surgery.

**Results:**

A total of 2216 trauma patients presented during the study period. Of 2216 patients, 224 (224/2216, 10.10 %) had complications reported at PI meetings; of these, 31 patients (31/224, 13.8 %) had FTR. PI patients were more likely to be older (mean age 51.3 years, SE 1.58, vs 46.5 years, SE 0.51; *P*=.008) and have higher ISS (median 22 vs 8; *P*<.001), compared with patients without complications. Physician-attributable errors (odds ratio [OR] 2.82; *P*=.001), most commonly errors in technique, and nature of injury (OR 1.91; *P*=.01) were associated with higher levels of harm, whereas delays in diagnosis or intervention were not. Each additional factor involved increased level of harm (OR 2.09; *P*<.001) and nearly doubled likelihood of FTR (OR 1.95; *P*=.01).

**Conclusions:**

Physician-attributable errors in diagnosis, technique, or judgment are more strongly correlated with harm than delays in diagnosis and intervention. Increasing number of errors identified in patient care correlates with an increasing level of harm and FTR.

## Introduction

### Background

Performance improvement (PI) is a key component of trauma center operations and centers on increasing patient safety through reduction of harm and iatrogenic error. According to the National Coordination Council for Medication Error Reporting and Prevention, harm is defined as “impairment of the physical, emotional, or psychological function or structure of the body and/or pain resulting therefrom.” [1]. The Institute of Medicine estimates that as many as 98,000 patients die in hospitals as a result of preventable medical errors each year [2]. Before this report, there was little uniformity in classifying and reporting postsurgical complications and levels of harm [3].

In 2009, the World Health Organization defined health care–associated harm as harm arising from or associated with provision of health care [4]. This distinguished health care–associated harm, which was potentially preventable, from harm related to underlying patient disease. The World Health Organization proposed a 1- to 5-point harm scale, ranging from no harm to death and including mild, moderate, and severe levels of harm; this was meant to standardize these definitions for safety and quality reporting. In 2010, the Agency for Healthcare Research and Quality (AHRQ) added the duration of harm to this 1- to 5-point scale, with permanent harm defined as harm with lasting effect of 1 year or greater and temporary harm defined as having effects lasting less than 1 year [5].

By defining the levels of harm, hospitals can better classify patient and provider errors that contribute to poor outcomes. Historically, hospital quality metrics included adverse occurrence rate and mortality rate. Failure to rescue (FTR) is an evolving quality metric. FTR represents the likelihood of a hospital to be unable to *rescue* patients from death after in-hospital complications [6]. As a measure of hospital response to complications, FTR has been studied for patients undergoing major elective surgeries [6,7] and has recently been applied to the trauma setting [8]. Studies have shown that FTR is a better marker for hospital quality than mortality rate or complication rate alone [6,7], and it has been shown to be the primary driver of differences in hospital quality for trauma patients [8]. Trauma centers with low overall patient mortality are more successful at rescuing patients who experience complications [9].

Previous studies in the trauma literature have explored the association between the error type and likelihood of posttrauma mortality [7,9-12]. However, these investigations have not focused on the examination and classification of other posttraumatic complications or potential errors. There are few studies in the literature that categorize the level of harm from a given complication using the new AHRQ guidelines. In addition, few studies have explored the effect of type and the number of human errors on posttraumatic complications, likelihood of FTR, and levels of patient harm using a standardized scale. Our study of specific human errors and their effect on patient complications provides a unique contribution to the existing literature on FTR.

### Objectives

We explored the factors associated with increased level of harm and FTR for trauma patients at a level 1 trauma center in New York City by examining reports from our weekly trauma PI records. Specifically, we sought to explore how different types of human errors and system errors contributed to the likelihood of patient complications and whether certain types of errors were more likely to cause patient harm than others. We also sought to analyze whether patient-related factors or physician-related factors were more likely to lead to patient harm. Finally, we were interested in discovering if an increasing number of patient- or physician-related factors contributed to a higher likelihood of patient harm from a given complication.

## Methods

### Patient Population

This was a retrospective study of trauma patients at Bellevue Hospital Center (BHC) in New York City. BHC is a large academic public hospital that is affiliated with NYU School of Medicine. The mean number of trauma patients admitted per year at BHC is 1500, with a 90:10 ratio of blunt to penetrating trauma [13]. Hospital catchment includes Manhattan and Western Brooklyn.

### Data Collection

Patient demographic information, including age, gender, ethnicity, insurance status, and physiologic information such as systolic blood pressure, Glasgow Coma Score (GCS), and injury severity score (ISS) on admission, was abstracted from the BHC trauma registry from January 1, 2016, to July 19, 2017. In addition, data were abstracted from weekly PI records over the given period.

### Identification of Harm Events

To assist with identification of as many complications/systems issues as possible, our program employs a PI coordinator who is an experienced physician extender, attends all trauma morning reports, and participates in daily walk rounds with the trauma service. We feel this allows us to capture adverse events in a timely fashion and ensure that they are reported to our PI meetings. All trauma surgeons in the department attend PI meetings and come to a group consensus for factors that contributed to a given complication. The discussions involve the entire trauma team but are led by the Trauma Medical Director. The Trauma Medical Director has taken a course focused on standardized PI Trauma Outcomes and Performance Improvement Course (TOPIC). The TOPIC incorporates the standardized definitions set forth by the AHRQ into its taxonomy classification.

The most commonly encountered complications in our institution are listed in our PI form, including deep vein thrombosis, pneumonia, chest tube–related complications, iatrogenic injury, death, and missed injury (Multimedia Appendix 1). A total of 7 factors contributing to these complications were examined, including delay in treatment, delay in intervention, error in diagnosis, error in technique, error in judgment, and nature of injury. *Nature of injury* was selected by attending surgeons if a patient’s underlying disease process (ie, severe medical comorbidities) or severity of injury contributed to a given complication. Multiple factors could be recorded for each complication. For patients who experienced multiple complications, only the most severe level of harm and its contributing factors were recorded. Physicians were also asked to rate the level of harm for each complication, using the standardized definitions set forth by the AHRQ (Multimedia Appendix 1) [5]. For simplicity of analysis, these ratings were then recoded into a 1- to 5-point scale, representing no harm, mild harm, moderate harm, severe harm, and unanticipated death.

### Statistical Analysis

Factors associated with FTR and level of harm were modeled using logistic regression, controlling for age, gender, Charlson score [14], ISS [15], need for surgery (ie, if patient required a surgery for trauma on index admission), error (in diagnosis, technique, or judgment), delay (in diagnosis or intervention), nature of injury, and total number of factors. Ordinal logistic regression was used to assess factors contributing to the increasing level of harm. Lipsitz goodness-of-fit test was performed for the ordinal level of harm regression, and Hosmer-Lemeshow test and receiver operating curves were performed for the FTR regression. Chi-square analysis was used to compare demographic and physiologic characteristics of patients presented at PI meetings with all other trauma patients over the given period. For quantitative variables, the Wilcoxon rank sum test was used to compare values. *P* values less than .05 were considered significant. All statistical analyses were performed using SPSS Statistics version 23 (IBM Corporation). This study received institutional review board approval.

## Results

### Patient Population

A total of 2216 trauma admissions presented during the study period. Of 2216 patients, 224 (224/2216, 10.10%) were presented at PI meetings. Of these, 31 patients (31/224, 13.8%) identified as FTR. Of the patients with complications, 81 (81/224, 36.1%) died during their admission. Of these mortalities, 52 (52/81, 64%) patients were classified as anticipated mortalities without opportunity for improvement (OFI), 12 patients (12/81, 14%) were classified as unanticipated mortalities with OFI, and 17 patients were classified as anticipated mortalities with OFI (17/81, 20%; Table 1).

**Table 1:** Complications for performance improvement patients (N=224).

		Patients, n (%)	Number of deaths
**Types of complication**			
	Abscess	3 (1.3)	0
	Deep vein thrombosis	17 (7.6)	9
	Pneumonia	5 (2.2)	2
	Clostridium difficile	3 (1.3)	1
	Postoperative bleeding	2 (0.9)	0
	Unplanned surgery	5 (2.2)	1
	Chest tube	12 (5.4)	0
	Iatrogenic injury	6 (2.7)	2
	Readmission	18 (8.0)	1
	Wound infection	11 (4.9)	4
	Missed injury	14 (6.3)	1
	Venous thromboembolism	11 (4.9)	1
	Sepsis	8 (3.6)	1
	Reintubation or unplanned intubation	13 (5.8)	0
	Triage issue	6 (2.6)	0
	Fall	8 (3.5)	0
	Dislodged tube	6 (2.6)	0
	Unplanned intensive care unit admission	6 (2.6)	0
	Others	44 (19.6)	22
**Deaths**			
	Deaths (including discharge to hospice)	84 (37.5)	—^a^
	Unanticipated mortality with opportunity for improvement	12 (14.8)	—
	Anticipated mortality with opportunity for improvement	17 (20.9)	—
	Anticipated mortality without opportunity for improvement	52 (64.1)	—
Failure to rescue, n (%)		31 (13.8)	N/A^b^

^a^Already mentioned.

^b^Not applicable.

### Factors Associated with Complications

The most common factor associated with a complication was nature of injury (92/224 patients, 41.1 %), followed by delays in intervention (41/224 patients, 18.3%). Moreover, 86 (86/224, 38.4%) patients with complications were described to have a mild level of harm associated with their complication, and 122 (122/224, 54.4 %) patients had only 1 factor associated with a given complication. The median level of harm associated with a given complication was 1 (intraquartile range [IQR], 0-4; Table 2).

**Table 2:** Factors contributing to complications and level of harm in performance improvement patients (N=224).

Factors contributing to complications		Value
**Type of factor, n (%)**		
	Delay in diagnosis	35 (15.6)
	Delay in intervention	41 (18.3)
	Error in diagnosis	5 (2.2)
	Error in technique	24 (10.7)
	Error in judgment	22 (9.8)
	Patient refusal	2 (0.9)
	Nature of injury	2 (0.9)
**Factor severity, n (%)**		
	No harm	70 (31.3)
	Mild harm	86 (38.4)
	Moderate harm	42 (18.8)
	Severe harm	12 (5.4)
	Death	14 (6.3)
Level of harm, median (intraquartile range)		1 (0-4)
**Number of factors per complication, n (%)**		
	1	122 (54.5)
	2	32 (14.3)
	3	8 (3.6)
	4	2 (0.9)

### Regression Models

In our logistic regression model for level of harm, physician-attributed errors (odds ratio [OR] 2.82; *P*=.001), most commonly errors in technique, were associated with higher levels of harm, whereas delays in diagnosis or intervention were not significant in this analysis. Nature of injury was associated with higher level of harm (OR 1.91; *P*=.01), whereas need for surgery was associated with decreased level of harm (OR 0.53; *P*=.02). Patients with higher ISS on admission (OR 1.04; *P*<.001) were more likely to have FTR. Each additional factor involved increased level of harm (OR 2.09; *P*<.001) and nearly doubled likelihood of FTR (OR 1.95; *P*=.01). Lipsitz goodness-of-fit test for the level of harm model demonstrated a *P* value of .26 (
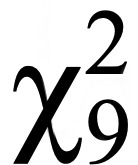
=49.5). For the FTR model, AOC SE was 0.048 (95% CI 0.71-0.9; *P*<.001), and Hosmer-Lemeshow chi-square value was 11.3 with a degree of freedom of 9 (*P*=.18; Table 3).

**Table 3:** Logistic regression model for level of harm and failure to rescue.

Covariates	Level of harm		Failure to rescue	
	OR^a^ (95% CI)	*P* value	OR (95% CI)	*P* value
Age	1.0 (0.9-1.0)	.59	1.0 (0.9-1.0)	.72
Male	1.0 (0.6-1.7)	.99	0.7 (0.3-1.7)	.43
Charlson score	1.0 (0.8-1.1)	.68	1.3 (0.9-1.7)	.07
Delay	1.4 (0.8-2.6)	.20	0.8 (0.3-2.3)	.69
Error	2.8 (1.5-5.2)	.001	1.9 (0.6-5.5)	.23
Nature of injury	1.9 (1.1-3.3)	.01	1.8 (0.7-4.6)	.21
Number of factors	2.1 (1.5-2.9)	<.001	1.9 (1.2-3.3)	.01
Any surgery	0.5 (0.3-0.9)	.02	0.9 (0.4-2.4)	.89
Injury severity score	1.0 (0.96-0.99)	.01	1.0 (1.02-1.07)	<.001

^a^OR: odds ratio.

### Patient Characteristics

Patients presented at PI meetings were more likely to be older (mean age 51.3 years, SE 1.58, vs 46.4 years, SE 0.51; *P*=.002). In addition, this group was more likely to present with lower GCS (median 14, IQR 3-15, vs median 15, IQR 15-15; *P*<.001) and higher ISS (median 22, IQR 11.75-29, vs median 8, IQR 4-10; *P*<.001). A higher proportion of patients presented at PI meetings were hypotensive on admission (7.1% vs 0.8%; *P*<.001). Finally, patients presented at PI meetings had a greater dispersion in Revised Trauma Score than patients without complications (median 5.64, IQR 1.02-5.64, mean 4.16, vs median 5.64, IQR 5.64-5.64, mean 5.5; *P*<.001). Trauma ISS was also lower in patients with complications (median 0.7, IQR 0.15-0.9, mean 0.56, vs median 0.94, IQR 0.84-0.97, mean 0.89; *P*<.001; Table 4).

Of those patients presented at PI meetings, the most common mechanism of injury was falls (102 patients, 102/224, 45.5 %). Moreover, 97 (97/224, 43.3 %) patients in this group were admitted to a step-down unit from the trauma bay, and 122 patients (122/224, 54.4 %) underwent a procedure during their admission (Table 5).

**Table 4:** Patients’ demographics and physiological characteristics (patients presented at performance improvement versus other trauma patients).

Patient demographics			All patients (N=2216)	Patients with complications (n=224)	Patients without complications (n=1992)	*P* value
**Age (years)**						
	All age, mean (SE)		49.6 (22.9)	51.3 (1.58)	46.5 (0.51)	.01
	Elderly patients (>65), n (%)		484 (21.84)	61 (27.2)	423 (21.23)	.04
**Gender, n (%)**						
	Male		1594 (71.93)	158 (70.5)	1436 (72.08)	.62
**Race, n (%)**						
	Asian		168 (7.58)	26 (11.6)	142 (7.12)	.02
	Black		380 (17.14)	24 (10.7)	356 (17.87)	.01
	Other		864 (38.98)	84 (37.5)	780 (39.15)	.63
	Unknown		4 (0.18)	0 (0.0)	4 (0.20)	.50
	White		800 (36.10)	90 (40.2)	710 (35.64)	.18
**Insurance status**						
	Private, n (%)		946 (42.68)	89 (39.7)	857 (43.02)	.34
	Public, n (%)		881 (39.75)	90 (40.2)	791 (39.70)	.89
	Self-pay, n (%)		389 (17.55)	45 (20.1)	344 (17.26)	.29
	Charlson score, median (IQR^a^)		1 (0-2)	1 (0-4)	1 (0-2)	<.001
	Intensive care unit, length of stay (days), median (IQR)		0 (0-0)	1.77 (0-5.9)	0 (0-0)	<.001
	Vent days, median (IQR)		0 (0-0)	0 (0-2)	0 (0-0)	<.001
	Revised trauma score, median (IQR)		5.6 (5.6-5.6)	5.6 (1.0-5.6)	5.6 (5.6-5.6)	<.001
	Trauma and injury severity score (N=2169), median (IQR)		0.9 (0.8-0.9)	0.7 (0.1-0.9)	0.9 (0.8-0.9)	<.001
**Physiologic characteristics (n=170)**						
	**SBP^b^ (mm Hg)**					
		All patients, median (IQR)	136 (112-155.2)	134.5 (115.2-155)	136 (122-152)	.18
		Hypotensive (SBP <90), n (%)	31 (1.46)	16 (7.1)	15 (0.75)	<.001
	**GCS^c^ (N=122)**					
		All patients, median (IQR)	15 (15-15), 14.1	14 (3-15), 10.67	15 (15-15), 14.48	<.001
		GCS<8, n (%)	124 (5.86)	70 (31.2)	54 (2.71)	<.001
	**ISS^d^ (N=1947)**					
		All patients, median (IQR)	8 (4-12)	22 (11.7-29)	8 (4-10)	<.001
		ISS>15, n (%)	376 (16.96)	150 (66.9)	226 (11.34)	<.001

^a^IQR: intraquartile range.

^b^SBP: systolic blood pressure.

^c^GCS: Glasgow Coma Scale.

^d^ISS: injury severity score.

**Table 5:** Injuries and procedures for patients presented at performance improvement (N=224).

Injuries and procedures		Value
**Abbreviated injury score, median (intraquartile range)**		
	Head	3 (0-4)
	Chest	0 (0-3)
	Abdomen	0 (0-1.7)
	Extremity	0 (0-2)
**Mechanism of injury, n (%)**		
	Stab	8 (3.6)
	Gunshot wound	11 (4.9)
	Assault	8 (3.6)
	Motor vehicle collision	9 (4.0)
	Bicycle	14 (6.3)
	Fall	102 (45.5)
	Pedestrian struck	44 (19.6)
	Motorcycle	2 (0.9)
	Others	26 (11.6)
**Disposition from trauma bay, n (%)**		
	Step-down unit	97 (43.3)
	Operating room	35 (15.6)
	Intensive care unit	54 (24.1)
	Monitored bed	24 (10.7)
	IR^a^	1 (0.4)
	Floor	12 (5.4)
**Procedure, n (%)**		
	Craniotomy	11 (4.9)
	Open reduction and internal fixation	29 (12.9)
	Exploratory laparotomy	20 (8.9)
	None	102 (45.5)
	IR	2 (0.9)
	Amputation	6 (2.7)
	Thoracotomy	6 (2.7)
	Intracranial pressure monitor	7 (3.1)
	Chest tube insertion	14 (6.2)
	Vascular surgery	4 (1.7)
	Debridement or washout	7 (3.2)
	Other	16 (21.4)

^a^IR: interventional radiology.

## Discussion

### Principal Findings

Care of trauma patients represents an environment that is prone to error. This is because of inherent illness of patients, time-sensitive decision making, and extensive handoffs and interplay of multiple specialties providing patient care. This was evident in this study as 51 errors contributed to a 8.93% complication rate (198 of 2216 patients) and 3.79% overall mortality rate (84 of 2216 patients) during the study period. Our reported mortality is in line with the 2% to 29% mortality that has been documented previously in the trauma literature [16,17]. Although reporting of complications is invariably center dependent because of the need for self-tracking and lack of consistent definitions, the rate of complications seen during our study period is also consistent with what has been reported [18,19]. To our knowledge, this is the first study in the trauma literature that focuses on factors that contribute to harm using the AHRQ system. In this study, increasing number of factors involved was significantly associated with increasing levels of patient harm. This is unsurprising, as the *Swiss cheese* model for error has shown that it is often multiple errors, not 1 single factor, that lead to harm for a given complication [20]. As trauma involves a complex interaction of clinicians and systems providing care, human factors inevitably affect the course of a critically injured patient. However, not all factors are created equal; in this study, certain errors were more likely to cause harm than others. Delay in diagnosis or intervention, for example, was not associated with a statistical increased level of harm or increased likelihood of FTR, whereas physician errors, either in diagnosis, technique, or judgment, were associated with increased harm (adjusted OR [AOR] 2.82, CI 1.52-5.25; *P*=.001). Similar provider errors have been described in preventable and potentially preventable deaths in the early resuscitation period and surgical intensive care unit, particularly when managing unstable patients, hemorrhagic shock, and threatened airways at major US trauma centers [10-12].

Several studies in the literature have previously linked patient-related factors with complications after traumatic injury. Bell et al demonstrated that preexisting comorbidities contributed significantly to mortality after complication in a trauma population [21]_,_ whereas others have shown that insurance status [22] and age [23] are associated with increased likelihood of FTR. In our analysis, we demonstrated that physician-related factors are more strongly associated with an increased risk for harm compared with underlying patient and injury attributes. We also demonstrate that error compounding significantly contributes to harm after complications with each increase in number of factors, effectively doubling the level of harm (AOR 2.09, 95% CI 1.52-2.91).

FTR represents a hospital’s inability to rescue a patient from complications. The FTR rate in our patient population was 13.8% (31/224). This is consistent with the literature, which cites FTR rates ranging from 6.8% to 19.8% [6,24,25]. Studies have examined specific factors that contribute to FTR and patient harm. Joseph et al [24] found that patients’ age, trauma mechanism, insurance status, and number of blood products administered on the second day of hospitalization significantly contributed to likelihood of FTR. Bell et al [21] found that uninsured patients had the lowest likelihood of developing a complication (OR 0.86), and yet they were more likely to experience FTR (OR 1.34) than patients who were privately insured (OR 1.25) or publicly insured (OR 1.17). Another study demonstrated that hospital- and physician-based factors, such as anesthesia board certification and presence of surgical house staff, were associated with FTR, whereas severity of illness was not [6]. Our analysis only demonstrated 2 significant factors for FTR in our regression model, although this may have been limited by sample size. Increased number of errors was associated with an almost 2-fold increase in FTR (AOR 1.95, 95% CI 1.16-3.27). ISS was the only patient-intrinsic factor identified and associated with a 4% increase per point increase in ISS (AOR 1.04, 95% CI 1.02-1.07).

Other findings, which are counter intuitive, are the inverse association between increasing ISS and need for immediate operative intervention and level of harm. One would infer that patients who required a surgery for their injuries had more severe injuries or were more critically ill, yet this population had a lower associated level of harm from a given complication. This suggests that, perhaps, there is a higher level of vigilance in this group of patients compared with those less injured or that physician-attributable errors significantly increased odds of increased level of harm more so than patient-based *nature of injury*. This suggests that there may be more opportunities for overall harm prevention.

### Limitations

There were several limitations present in this study. As this study was retrospective in nature, it was not designed to prove causality between the relationships and associations between harm and outcomes demonstrated. We are only able to make generalizations based on the experiences of a single trauma center. Reporting of complications is voluntary and lacks uniformity; however, we believe that we are fortunate to have a PI coordinator who is invaluable in assisting the trauma service in identifying as many complications and adverse events as possible. The level of training of the individuals making the error or service responsible for the error was not captured; this highlights the difficulty in assigning attribution for postinjury complications. This study was also limited by a small sample size of patients, especially patients who were deemed FTR and may predispose to type II error. It would have been ideal to capture where in the course of evaluation or postinjury course that complications occurred (ie, resuscitation, operating room, or acute care), but this information was not available. In addition, physicians were asked to record factors contributing to harm, and these may be subjective measures, even if reached by consensus. Finally, it is possible that our analysis may be affected by hindsight bias. Surgeons at our institution may have been more likely to find more contributing factors or errors with a poor outcome such as a mortality or serious complication when reviewing the case in PI meetings. Unfortunately, this is a limitation of this retrospective case review.

### Conclusions

This analysis applies the current concept of FTR and patient complication prevention to the trauma patient population. We have demonstrated that the increasing number of errors identified in patient care directly correlates with level of harm seen after traumatic injury. Interestingly, certain types of errors are more associated with harm; in particular, physician-attributable errors are more strongly correlated with harm than underlying patient factors.

This is, to our knowledge, one of the first studies to categorize level of harm using new AHRQ guidelines. Future studies should examine interventions that could prevent or mitigate physician-attributable errors. These could be further classified into error type (ie, skill-, rule-, or knowledge-based) to further assess which errors carry more weight or risk to patient harm. This information could then be used to develop further provider training or trauma system enhancement for quality improvement. Prospective evaluation of these specific interventions could then be used to assess their impact on patient-related complications and levels of harm.

## Appendix

Multimedia Appendix 1 Performance improvement form.

## Abbreviations

AHRQ: Agency for Healthcare Research and Quality
AOR: adjusted odds ratio
BHC: Bellevue Hospital Center
FTR: failure to rescue
GCS: Glasgow Coma Scale
IQR: intraquartile range
ISS: injury severity score
OFI: opportunity for improvement
PI: performance improvement
TOPIC: Trauma Outcomes and Performance Improvement Course
