# Supplementary material for: Postinjury Complications: Retrospective Study of Causative Factors
Source: JMIR Hum Factors. 2019 Sep 26;6(3):e14819. doi: 10.2196/14819 (PMC6787527; doi:10.2196/14819)
Supplement: Multimedia Appendix 1 [file 14819-305897-1-SP-dg.pdf]

Appendix: PI Form

Attending M&M Case Review (Level 1 Review)

**Complication (check all that apply):**				
☐Abscess	☐C diff colitis	☐Chest-tube related	☐Death	☐Decubitus
☐DVT	☐Evisceration	☐Iatrogenic	☐Missed injury	☐PE
☐Pneumonia	☐Post-op bleeding	☐Readmission	☐Sepsis	☐Unplanned OR return
☐UTI: Foley catheter Choose an item.		☐Wound infection	☐Other: Click here to enter text.	

**Contributing Factors:**	**Recommended Action:**	**Level of Harm:**
☐ Delay in diagnosis	☐ Systems enhancement	☐ 0 – No harm, no detectable harm
☐ Delay in intervention	☐ Counseling	☐ 1 – Potential for harm
☐ Error in diagnosis	☐ Credentialing action	☐ 2 – Mild temporary harm
☐ Error in technique	☐ Education	☐ 3 – Mild permanent harm
☐ Patient refusal	☐ Guideline/Protocol	☐ 4 – Moderate temporary harm
☐ Error in judgment	☐ None	☐ 5- Moderate permanent harm
☐ Nature of disease/injury	☐ Refer to TPRC	☐ 6- Severe temporary harm
☐ None/Care was standard	☐ Periodic reporting	☐ 7 – Severe permanent harm
☐ Other: enter text here	☐ Other: enter text here	☐ 8– Unanticipated Death
	☐ Refer to: enter text here	

Discussion / Recommendations for next time:

Discussion:
Click here to enter text.

Recommendations:
Click here to enter text.

Action Plan:
Click here to enter text.

Case Status: Choose an item. **Name:** Choose an item. **Date:** Enter date.
